# Responsibly shaping technology innovation for the energy transition: an RRI indicator system as a tool

**DOI:** 10.3389/frma.2023.1157218

**Published:** 2023-06-29

**Authors:** Tobias Buchmann, Patrick Wolf, Matthias Müller, Marion Dreyer, Frank Dratsdrummer, Bianca Witzel

**Affiliations:** ^1^Department of System Analysis, Zentrum für Sonnenenergie- und Wasserstoff-Forschung Baden-Württemberg, Stuttgart, Baden-Württemberg, Germany; ^2^Department of Innovation Economics (520i), University of Hohenheim, Stuttgart, Germany; ^3^Department of Economics, University of Insubria, Varese, Italy; ^4^Flying Faculty, Department of Economics, Turkish-German University, Istanbul, Türkiye; ^5^DIALOGIK gemeinnuetzige Gesellschaft für Kommunikations- und Kooperationsforschung mbH, Stuttgart, Baden-Württemberg, Germany

**Keywords:** Responsible Research and Innovation (RRI), indicator system, renewable energy innovation, innovation diffusion, innovation acceptance

## Abstract

Efforts to reduce global greenhouse gas emissions have had limited success. For many, the hopes rest on new energy innovations to advance the energy transition process. In this paper, we develop a Responsible Research and Innovation (RRI) base indicator system to steer the design of innovations in the field of energy transition innovations and, thus, improve social acceptance of these innovations. We propose a guideline for its application to assist R&D performing organizations and funding organizations in the design, selection, and communication of research proposals. The indicator system is intended to promote early integration of environmental and social aspects, support the formation of teams aware of the different responsibility aspects of innovation, and monitor progress in regard to relevant RRI dimensions.

## 1. Introduction

Political efforts have led to only limited reductions in global greenhouse gas emissions. Accordingly, many hopes rest on energy transition innovations that are effective, experience a high level of social acceptance and can thus be implemented widely in order to successfully advance the energy transition process. In our study,^1^ we refer by social acceptance to the passive or active socio-political and community acceptance of energy technologies, i.e., the public's passive or active approval (based on subjective valuation rather than scientific expertise) of decisions by others (Bertsch et al., 2016). A starting point of our considerations is the fact that passive or active social acceptance of energy transition innovations cannot be taken for granted, despite the general broad acceptance of the transition of the energy system. One reason for this is that potential negative impacts of innovations are often only recognized, regulated and mitigated, after the diffusion of the innovation and thus after they have negatively affected the acceptance of customers (Collingridge, 1980; see also Müller, 2016; Schlaile et al., 2018 for a discussion). As a result, research, technology development and innovation (RTI) processes are often not forward-looking, and there is a lack of early integration of environmental and social research.

Despite an ongoing debate about the normative aspects of innovations (see for example Schlaile et al., 2017) innovations are often considered as something inherently positive and a determining factor of economic growth and long-term prosperity. However, there is increasing recognition that innovations can have undesirable consequences, such as environmental destruction, impairment of human rights, negative employment effects, or undesirable distributional effects (Giuliani, 2018) that delimit their societal benefits and social acceptance. Innovation is hence neither inherently good nor self-regulatory. This is also recognized in most of the literature on Responsible Research and Innovation (RRI). As also (Koops, 2015, p. 2) puts it: “although ‘responsible innovation' is a term that is increasingly used both in academic and in policy circles, it is by no means clear what exactly the term refers to, nor how responsible innovation, once we know what is meant by this, can or should be approached.” In other words, while the central idea of RRI—to adapt innovations to societal wishes and needs—is relatively unchallenged in the innovation and policy research community, beyond existing methods such as technology assessment or foresight, many questions regarding the actual application of the RRI concept in a policy, research, and business context are unanswered.

Recently, research projects have delved more deeply into the question of how RRI can be applied as a toolkit to help policy makers, scientists, and businesses (e.g., see https://rri-tools.eu/or and https://www.v4innovate.de/). Guidelines and indicator systems are being developed and applied as an orientation for the development and diffusion of more (societally and environmentally) responsible technologies (e.g., Wickson and Carew, 2014; Meijer and van de Klippe, 2020). Traditionally, RRI has been applied to potential breakthrough (and/or general purpose) technologies such as biotechnology, genetic engineering, and quantum computing (Owen, 2019). However, there is a role for RRI not only for specific technologies but also for complex socio-technical transformation processes (including a wide range of different technologies) such as the transition of the energy system toward carbon neutrality (Owen et al., 2013).

In this paper, we set out a concept for an RRI indicator system that can help to cope with some of the key challenges arising when applying an RRI approach based on the example of technical innovations for the energy transition. We highlight three challenges, namely how to deal with the normativity of the RRI concept, how to handle subjectivity along the evaluation process and how to take the specificity of energy technology innovations into account. The foreseen users of the indicator system are R&D funding organizations (government research funding and funding from private foundations) and R&D performing organizations (public and private). The indicator system will help R&D funding organizations to: Find inspiration for funding anticipatory research on newly emerging energy transition technologies; Identify RRI-relevant research needs and research gaps in regard to already existing energy transition technologies and set up corresponding research funding programs (e.g., with the help of expert groups and/or multi-stakeholder agenda-setting processes informed by the indicator system); Inform the selection of research proposals for funding and take well-grounded funding decisions; Help communicate decision-making on proposal selection in a transparent manner.

The remainder of this paper is structured as follows: In Section 2, we present the conceptual background of the RRI indicator system. Section 3 describes the development of RRI process and product indicators. In Section 4, we delineate some concluding remarks.

## 2. Conceptual background

### 2.1. The need for RRI

The perception of the RRI concept varies widely in the literature. For example, some authors see RRI as a current concept (de Jong et al., 2015), others as an approach (Blok and Lemmens, 2015), ideal or desire (Owen et al., 2013), or process (Stilgoe et al., 2013; von Schomberg, 2013). Still others see RRI only as hype (de Jong et al., 2015) or even as a (failed) experiment (Delvenne, 2017). One central reason for this is the problem of how to actually apply the RRI concept. RRI builds on various precursor concepts that date back to the middle of the last century, both at the level of research funding policy and at the scientific level. In this regard, various streams within Science, Technology, and Society Studies (STS) are often cited in the literature as the origin of RRI (van Oudheusden, 2014; Koops, 2015; Carbajo and Cabeza, 2018; Brundage, 2019). Arguably, RRI has the most common ground and overlap, especially in scholarly discourse, with the concept of technology assessment (TA; see, for example, Grunwald, 2014; van Oudheusden, 2014; van Lente et al., 2017; Matthews et al., 2019) and the differences and similarities between these two concepts remain the subject of ongoing discussions.

Generally speaking, RRI processes involve activities that go beyond a mere compliance with legal rules. They refer to sub-legal responsibilities and aim at voluntarism, self-commitment and compliance with sub-legal rules such as administrative provisions (e.g., funding regulations). In fact, the legal framework conditions must be complied with anyway, e.g., minimum distances when operating wind turbines. However, it can also be the case that laws (abroad) are generally felt to be too weak, e.g., in the field of cobalt extraction in Congo and the resulting problems with the acceptance of batteries (see for example Arnaldi and Gorgoni, 2016). Another important reason for the need of RRI is that regulation (and standardization) tends to lag behind the development of innovations. Thus, RRI is especially valuable when new technologies are not yet regulated or when technology development is still ongoing. Its application can contribute to realizing a more transparent research and innovation process, to increasing accountability and creating trust.

As emphasized by several authors, the aim of an RRI-informed research and innovation process consists of “shaping innovation” instead of “shaping technology” only (see for example Grunwald, 2011; van Lente et al., 2017). In our study, we relate RRI to the development of innovations (e.g., a new energy storage) and the further development of existing innovations (e.g., new types of batteries) for the energy transition. We have a similar broad understanding of the notion of innovative products as Van de Poel et al. (2017) to whom a product is “any kind of output that can be used by another actor for another end.” Moreover, the innovation needs to be the outcome of a deliberate knowledge generation process, which is typically an R&D activity. A central challenge here is that innovation processes are characterized by a high degree of uncertainty regarding their outcome, so-called “true uncertainty” (Knight, 1921) and a partly, non-linear and non-deterministic outcome. At the same time, it is clear that innovation is not a totally arbitrary process (like “manna from heaven”). Accordingly, the possibilities of early and exact planning and assessment of technology impacts are limited, yet, we firmly believe that it is possible to design systems and processes in such a way as to increase the probability that an innovation process and its outcome will be socially desirable and accepted.

In similar vein, we have to acknowledge that innovation and the diffusion of innovation are not steps isolated from society. Rather, these steps take place in a systemic context and in interaction with societal actors and ideas. The energy transition generally, and this includes the development and use of energy technologies, is a transformation process involving society as a whole (Schneidewind, 2018) and which is characterized by a high degree of complexity, uncertainty and ambiguity. Following the idea of Owen et al. who state: “The aim of RRI policy is that research and innovation should have a societally beneficial impact” (Fitjar et al., 2019, p. 773), we aim to operationalize RRI in form of a tool for directing the energy transition into societally beneficial directions. However, we abandon the idea that there is such a thing as an optimal solution to the energy transition or societal challenges in general and enhance the likelihood that energy transition technologies contribute to addressing other grand social challenges (e.g., loss of biodiversity; Schlaile et al., 2017).

### 2.2. RRI as a tool

Soon after the introduction of the RRI concept in the context of the European research agenda, discussions began on how the concept, which was initially discussed mainly theoretically, could be used in practice and for this purpose operationalized and possibly even made measurable (Monsonís-Payá et al., 2017). In this regard, Wickson and Carew (2014) argue that the formulation of both quality criteria and tangible RRI indicators in particular is an essential necessity if RRI approaches are practically applied in science, science funding, by innovation drivers and further stakeholders in the research and innovation system. The need for instruments to implement the RRI concept was also seen by the European Commission and led to projects to create indicators with a focus on monitoring RRI (European Commission, 2015; European Commission et al., 2018).

Despite these initial efforts and the increasing recognition of the necessity of RRI operationalization in the scientific community, corresponding (further) developments initially remained sporadic. Iatridis and Schroeder (2016) stated the development of tools and metrics in the RRI context was still in the development phase. Recent publications with reference to the practical use of RRI indicate an increasing interest in this field of tools-oriented research (see e.g., Yaghmaei et al., 2019; Kwee et al., 2021). The volume by van de Poel (2020), for example, provides an overview of recent efforts to further developing indicators for the operationalization of the RRI concept. Today, there exist various indicator sets that vary significantly from one another in terms of their characteristics, goals, addressed RRI dimensions and/or types of assessment. As also van de Poel's (2020, p. 350) states “one might wonder how what is supposedly one concept can lead to such diverse constructs.”

When scanning the relevant literature, we found indeed that existing sets of indicators mostly stand for themselves and miss reference points between each other. While this can be explained at least partly by different objectives, it is also apparent when comparing indicators sets that have similarly oriented assessment approaches. To create a common basis for RRI indicators, (Wickson and Carew, 2014, p. 261) develop a set of RRI quality criteria covering aspects which “'good' science and ‘responsible' research and innovation should entail.” They further suggest that the criteria and their proposed measurement should be adapted to the respective project or evaluation task. Extended and further developed representations of these quality criteria were developed within the framework of the RRI Tools project (Kupper et al., 2015) and the implementation framework of Fraaije and Flipse (2020). These criteria aimed at providing a comprehensive overview of “characteristics of research and innovation practices that should be targeted in assessment, monitoring or (self-)evaluation tools” (Kupper et al., 2015, p. 17) and were specifically designed as an assistance to operationalization. So far, however, the corresponding quality criteria have hardly been used to create actual indicator systems.

There are a number of concept-immanent challenges when operationalizing RRI (see also the discussion by van de Poel, 2020). First, we have to acknowledge that RRI is a deeply normative concept. This normativity concerns not only the question what a responsible innovation or responsible research might be, but also what RRI is and can or should do (see also Doezema et al., 2019; Wittrock et al., 2021; Völker et al., 2023). As also van de Poel (2020, p. 351) states, RRI “expresses what is desirable, not what is factually the case.” This means that in the attempt to measure RRI, “at least some of the attributes need to be normative or involve normative judgments” (van de Poel, 2020, p. 351). At the same time, we see that there is no common agreement or understanding of what RRI is or should be. This links to the general problem of subjectivity when operationalizing RRI through indicators, both from the perspective of evaluators but also from the perspective of those being evaluated. Consequently, we think that it is problematic to use indicators to judge between good or bad RRI but rather to guide better design of innovations. Second, and closely connected to the problem of normativity and subjectivity there is the general problem of how to measure RRI or RRI processes. This problem is particularly elevated by the fact that RRI can be seen as a “complex matter that can be related to diverse contexts, subject areas, and actors” (Tassone et al., 2018, p. 346). This ultimately leads to the point that a corresponding indicator approach requires rather qualitative indicators, which, nevertheless, is associated with an increased subjectivity in the assessment and thus an increased reliability problem. Some indicator sets try to mitigate this problem by reverting to a binary measurement without the possibility to apply a more granular differentiation. Although this can indeed increase reliability by limiting the measurement options, it seems questionable whether such an approach can be justified regarding the complexity of the RRI concept. Finally, especially when applying RRI not only for single technologies but for complex socio-technical transformation processes (including a wide range of different technologies), the problem of specificity arises. Given the inherent normativity and subjectivity as well as the problems when measuring RRI, one may be tempted to apply generic indicators. Thereby, however, one risks to dilute the operationalization of RRI by using unprecise and unspecific indicators and by neglecting the specific and unique context of different technologies. In fact, it is highly likely that what can be considered an adequate concept for an indicator system will differ considerably across technologies but also for one single technology over time.

## 3. Indicator system conception

### 3.1. General structure of the indicator system

In the context of RRI, the literature differentiates broadly between two different perspectives. First, from a “product perspective,” RRI addresses directly the marketable products resulting from an innovation process through normative anchor points. Following the definition by von Schomberg (2013), these normative anchor points are (ethical) acceptability, sustainability and societal desirability. The product can be both a physical product and a non-physical product (e.g., in the sense of a service; Owen et al., 2013). Second, at a “process level,” RRI aims at a “more responsive, adaptive and integrated management of the innovation process” (von Schomberg, 2013). The process perspective can be subdivided into different process dimensions. Stilgoe et al. (2013) differentiate here between the dimensions of inclusion, reflexivity, anticipation and responsiveness. Based on an extensive review of the existing RRI literature, Fraaije and Flipse (2020) add transparency as a fifth dimension, as it has relatively broad support in the literature and shares various connections with the other dimensions. This subdivision into five dimensions is also taken up for the indicator system presented in this paper.

Although the distinction between product and process level was introduced early in the RRI literature, the focus mostly lies on the process level (Thorstensen and Forsberg, 2016). As also (Burget et al., 2017, p. 8) note: “researchers have seen RRI primarily as a process including stakeholders, anticipating, reflecting and responding to the needs and values of society.” (Wickson and Carew, 2014, p. 260) state in this regard that “any product developed through an innovation process will inevitably enter a complex web of interactions of use, the outcomes of which will be inherently uncertain” and that it would therefore make more sense to focus on the process side in the context of developing evaluation criterion for RRI. This is also underlined by Burget et al. (2017, p. 14), who conclude that “what the notion of RRI seems to point to is not to focus so much on bringing about certain outcomes as paying attention to the processual elements required for the implementation of a process.” However, this does not mean that the product perspective is to be completely neglected, but rather indicates that it is especially difficult to consider this level separately. In fact, the literature increasingly points out that a clear and sharp separation of the product and process side is hardly possible (Wickson and Carew, 2014; Thorstensen and Forsberg, 2016). This is highlighted by Wickson and Carew (2014, p. 260) also with regard to operationalization of RRI. They stress that in the context of an “[...] evaluation of a process of innovation, one still needs the capacity to also consider existing preconditions, envisaged products and engaged people, since all of these elements shape, guide and, to some extent, generate, and characterize the RRI processes [...].”

Consequently, the RRI indicator system presented here is focused on the process level while explicitly including the product level on the basis of so-called normative anchor points specified by von Schomberg (2013). These anchor points were also deliberately implemented because they reflect the “Grand Challenges” of our time (von Schomberg, 2013) and thus, are aspects that are essential to consider in context of energy transition.

### 3.2. Product perspective

According to von Schomberg (2013), the RRI product dimension captures products in relation to overarching and specific normative anchor points. In this regard, von Schomberg (2013) distinguishes three overarching indicator groups that need to be considered: Ethical acceptability, sustainability and social desirability. We draw on these central anchor points, also because they are reflected in the product-level subdivisions of most existing publications (see e.g., European Commission, 2015; Stahl et al., 2017; Fraaije and Flipse, 2020). Ethical acceptability of RRI products is reflected especially with regard to the fundamental values of the EU Charter of Fundamental Rights (von Schomberg, 2013). The essential personal freedoms and rights described in the Charter can be divided into six sub-areas: Dignity, Freedoms, Equality, Solidarity, Citizen's rights and Justice. Following this subdivision, research and innovation should strive for products that comply with the values of human dignity, which is expressed, among other things, in the integrity of the person and the exclusion of forced labor (especially in product production or service provision). In addition, the values of freedom have to be respected, which refers in particular to respect for private life, the right to freedom and security, and the protection of personal data. According to the principle of equality, general non-discrimination, respect for cultural, religious and linguistic diversity and equality between men and women are essential rights. The values of solidarity encompass numerous aspects, of which the prevalence of fair and equitable working conditions and the observance of youth labor protection are particularly relevant to the product dimension.

von Schomberg (2013) suggests sustainability as the second overarching normative anchor point of RRI which requires research and innovation to strive for the product's alignment with the EU's objective of sustainable development. This objective is subject to a 3-fold division of sustainability, analogous to the definition of sustainable development by the United Nations: economic sustainability, social sustainability, and environmental sustainability. Building on these three pillars, sustainable development aims at the capacity for “meeting the needs of the present whilst ensuring future generations can meet their own needs” (European Commission, 2014). Environmental sustainability is concerned with the extent to which the technology to be developed allows or hinders the considerate use of natural resources. Social sustainability focuses primarily on social issues, such as whether the development will lead to irreversible changes that future generations might not want. Finally, economic sustainability emphasizes that the way of doing business must not damage the required resources in the long term, so that they are also available in at least the same quality in the long term.

The third overarching normative anchor point is social desirability, which can essentially be represented by the normative anchor points of the Treaty on the EU. Some of the relevant aspects here can already be found in the fields of ethical acceptability and sustainability. Social desirability emphasizes that responsible technology development should not only be about developing technologies with low physical risk for people and the environment. In addition, a broad and inclusive deliberative process should be used to discuss which innovations would or would not be desirable for other reasons in solving major societal problems (in our case clean and safe energy plus climate protection). Such reasons can be, for example, the degree of social inclusion or the degree of fair benefit/burden sharing that can be achieved with a technology (Sarewitz and Nelson, 2008).

The superordinate anchor points ethical acceptability, sustainability and social desirability are subdivided into subordinate anchor points (Table 1). The product-oriented anchor points are integrated into the process dimension. This builds on von Schomberg's (2013) RRI concept according to which products should be designed with regard to their normative anchor points and thus the anchor points should by definition already be considered and included in the R&D process.

**Table 1:** Anchor points related to the RRI product level.

**Superordinate anchor points**	**Subordinate anchor points**
Ethical acceptability	Compatible with human dignity rights
	Compatible with rights of freedom
	Compatible with rights of equality
	Compatible with rights of solidarity
Sustainability	Ecologically sustainable
	Socially sustainable
	Economically sustainable
Social desirability	Social justice
	Protection of environment and health
	Influence on quality of life
	Promotion of scientific/technical progress

### 3.3. Process level

The design of indicators addressing the process level is based on the dimensions of inclusion, reflexivity, anticipation and responsiveness formulated by Stilgoe et al. (2013), as well as the fifth dimension of transparency proposed by Fraaije and Flipse (2020). In addition to these general process dimensions, various quality criteria are formulated in the literature (Wickson and Carew, 2014; Kupper et al., 2015; Fraaije and Flipse, 2020), on the basis of which a further subdivision of the process dimensions becomes possible. These quality criteria represent essential characteristics of research and innovation practices that should be considered in monitoring, assessment, or (self-) evaluation tools in the RRI context (Kupper et al., 2015). For this reason, we use these quality criteria as a basis for developing and formulating the process indicators assigned to the various dimensions. Based on a review of literature, we first collected existing RRI quality criteria. Here, in particular, the extensive work of Wickson and Carew (2014), Kupper et al. (2015), and Fraaije and Flipse (2020) already provides a large number of more than 100 criteria. In a second step, the full list of criteria was reviewed and bundled to serve as indicators in order to reduce the multitude of quality criteria to a manageable number. The associated (re)arranging, (re)interpreting, and/or applying of existing quality criteria is also explicitly desired in the context of RRI operationalization (Wickson and Carew, 2014). In this way, indicators can be derived from the RRI dimensions that cannot be measured directly, which make the respective RRI dimension measurable as a proxy (van de Poel, 2020).

While the challenges of operationalizing RRI as discussed above cannot be completely prevented, they can at least be reduced through an assessment process that is guided and that follows an assessment framework that includes a clear rubric, which guides the evaluator through the evaluation process by verbalizing the different indicator levels. A rubric, as for example shown in Wickson and Carew (2014), can reduce the subjectivity of assessments in this context (van de Poel, 2020) and at the same time is an appropriate way of evaluating the quality of RRI and the associated progress by clarifying explanations and providing “inspiration, concrete guidelines, and direction for improvement” (Wickson and Carew, 2014, p. 263).

The rubric for the indicator system presented in this paper is based on a four-scale classification, which reflects the degree of (methodological) consideration of the individual indicator fields and is thus intended to reveal opportunities for improvement. The bottom scale classification “low” therefore shows that the corresponding RRI aspect is not or only slightly pronounced, while “very high” indicates that the aspect is expressed very well. While the various RRI dimensions should be considered for each technology or use case, the optimal level can vary. van de Poel's (2020, p. 351) illustrates this with the example of the inclusion dimension, stating that “the ‘right” level of stakeholder involvement may not be the same for every innovation, or for each technical domain.” Especially for the field of energy system transformation, which in its breadth encompasses numerous different technical domains and innovations, such differences cannot be avoided. In view of this problem and the chosen level of observation, the indicators are not designed to represent a fixed level of responsibility for the individual dimensions. Instead, greater emphasis is placed on examining whether appropriate methodological approaches are integrated into or planned for the innovation process that allow for a specific process-internal assessment and integration of the RRI aspects.

The following sections deal with the individual process dimensions included in the indicator system and show the indicators formulated based on the quality criteria cited in the literature and the characteristics developed for them.

#### 3.3.1. Anticipation

Anticipation describes the systematic attempt to investigate and assess as best as possible the (intended or unintended) consequences of innovations as early as possible. A related question that probably best describes this dimension is the question “what if?” (Owen et al., 2013). It is important to note that anticipation does not make predictions, but can highlight possible alternative paths that might be overlooked without a targeted and systematic anticipatory approach. As such, anticipation serves a purposeful reflection on the meaning, purpose and possible impact of an innovation and the systematic comparison of alternative options (Owen et al., 2013). The methods used range from foresight, technology assessment, horizon scanning, scenario analysis, and vision assessment to science fiction. The optimal timing for the use of these tools is in tension with the Collingridge Dilemma (Collingridge, 1980). They need to be applied early enough to have an impact and at the same time late enough to be useful. The result of a successful anticipation process is a sound understanding of the dynamics and forces that determine future technologies (Borup et al., 2006; Stilgoe et al., 2013; Burget et al., 2017). Additionally, anticipation can directly support the realization of responsible products since products become more ethically acceptable by becoming more “resilient” (Stilgoe et al., 2013) and also “socially robust” (Van den Hoven et al., 2013).

**Figure 1:**
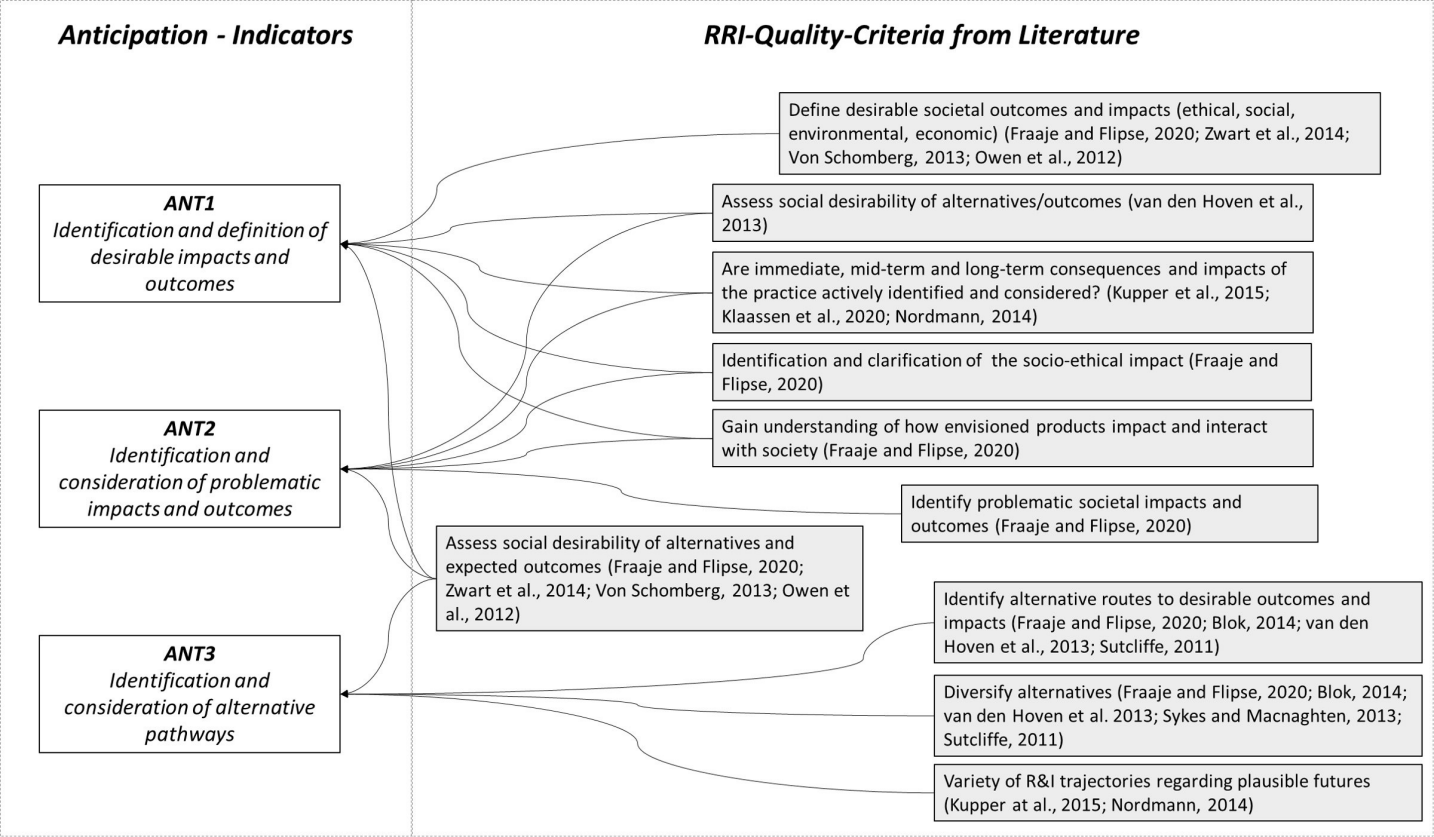
Quality criteria and indicators for anticipation.

The quality criteria identified in the literature, shown in Figure 1, can essentially be divided into three groups, which are also found in the qualifiers presented by Fraaije and Flipse (2020). Indicator ANT1 aims at the extent to which an identification and definition of the desirable impacts and outcomes is planned within the research process (Owen et al., 2012; von Schomberg, 2013; Zwart et al., 2014). Indicator ANT2 points at the opposite case with the verification of the implementation of an identification and consideration of problematic impacts and outcomes. A corresponding need to consider both aspects also results from the statement by Owen et al. (2012), according to which responsible innovation should consider both intended and unintended aspects of science and innovation. This dual consideration is often bundled in formulated quality criteria by generally asking for consideration of possible consequences or desirability. In this way, it should be considered whether there is generally a systematic attempt to identify and consider potential immediate to long-term effects (Nordmann, 2014; Klaassen et al., 2017). The focus of consideration is partly on the societal level with the socio-ethical impact (Fraaije and Flipse, 2020), the social desirability of the outcome (Van den Hoven et al., 2013) and the impact and interaction of the product with society (Fraaije and Flipse, 2020). However, other criteria with the identification of intended and unintended, long-term economic, environmental and social consequences and impacts of the practice (Nordmann, 2014; Kupper et al., 2015; Klaassen et al., 2017) call for a broader spectrum of consideration, which is also reflected in the anchor points formulated at product level. Finally, the third indicator (ANT3) addresses the issue that research processes should include the identification and consideration of alternative research, development and innovation paths. This covers a frequently cited aspect of anticipation, according to which alternative routes to the identified desirable outcomes (Sutcliffe, 2011; Van den Hoven et al., 2013; Blok, 2014; Fraaije and Flipse, 2020) or alternative R&I trajectories in general (Nordmann, 2014; Kupper et al., 2015; Klaassen et al., 2017) should also be identified or considered within the research process. Table 2 presents the indicators and the corresponding rubric descriptors. The descriptors are based on and extend the formulations presented by Wickson and Carew (2014).

**Table 2:** Rubrics for anticipation.

	**Low**	**Moderate**	**High**	**Very high**
**ANT1** Identification and definition of desirable impacts and outcomes	Assumption of desired impacts and outcomes, no clear efforts to explore possible future scenarios.	Informal attempts to future cast desirable impacts and outcomes at limited points in the project.	Inclusion of future casting activities concerning desirable impacts and outcomes in relation to the anchor points at some point during the R&I process.	Structured, targeted periodic analytical review of desirable impacts and outcomes in relation to the anchor points is foreseen.
**ANT2** Identification and consideration of problematic impacts and outcomes	A single optimistic prognosis for future project outcomes with no clear effort to identify risks or survey possible future scenarios.	Informal attempts to future cast problematic or unintended impacts and outcomes at limited points in the project.	Inclusion of future casting activities regarding problematic or unintended impacts and outcomes in relation to the anchor points at some point during the R&I process.	Structured, targeted periodic analytical review of problematic or unintended impacts and outcomes in relation to the anchor points is foreseen.
**ANT3** Identification and consideration of alternative pathways	A single optimistic prognosis for chosen path with no clear effort to identify alternative research, development and innovation paths.	Informal attempts to future cast alternative trajectories at limited points in the project.	Inclusion of future casting activities regarding alternative trajectories at some point during the R&I process.	Inclusion of formal processes of future casting at various points throughout the research and innovation process for alternative research, development and innovation paths.

#### 3.3.2. Reflection

Reflection means holding up the proverbial mirror to one's own activities, commitments and assumptions (Stilgoe et al., 2013). It is about continuously reflecting on the goals of RTI, as well as the decisions and actions taken to achieve them, being aware of the limits of knowledge and being mindful that a particular view of the issue is not necessarily universally accepted. It is important that one's own value system must also be subject to critical reflection as a yardstick for evaluation (second-order reflexivity; Stilgoe et al., 2013; Lubberink and Blok, 2017). Unlike a private (professional) self-criticism, reflection in the context of RRI is a public matter (Wynne, 2011) and thus always involves reference to an external value system (von Schomberg, 2013). Reflexivity is closely related to anticipation. The main difference is that anticipatory processes aim at identifying possible impacts/scenarios while reflexive processes aim at gaining a deeper understanding of the relevant processes creating these potential scenarios.

**Figure 2:**
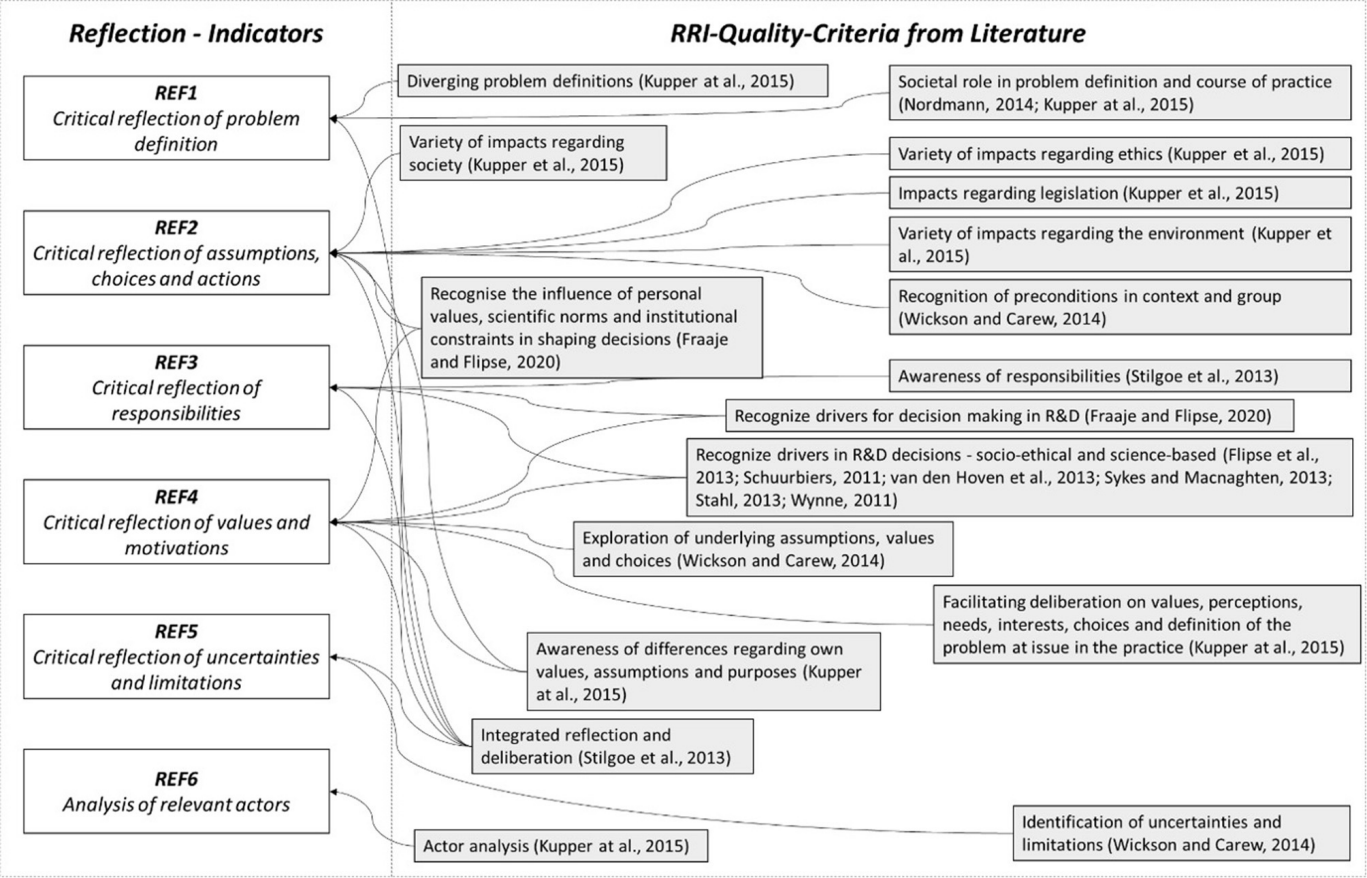
Quality criteria and indicators for reflection.

With regard to the dimension of reflection, numerous quality criteria have been formulated in connection with RRI which can be divided into six groups, as shown in Figure 2. Mapped by indicator REF1 is the critical reflection in relation to the problem definition. For example, Kupper et al. (2015, p. 30) here refer to the need to consider “potentially diverging problem definitions circulating and societal values informing such definitions” as an essential building block for in-depth reflection and investigation of the current situation (Nordmann, 2014; Kupper et al., 2015; Klaassen et al., 2017). In addition to the problem definition, the respective assumptions, choices and actions (Wickson and Carew, 2014) should also be subjected to critical reflection as part of the research process (indicator REF2). This is also underlined by the fact that reflective processes should generally help actors involved to “gain a deeper understanding of the social and ethical implications of their actions” (Fraaije and Flipse, 2020). Kupper et al. (2015) also describe this with the question of sufficiently addressing ethical, legal, social, and environmental aspects and/or impacts in the context of the FTI practice at hand. For reflection, it is also useful and necessary for the actors involved to become aware of their own responsibilities as well as accountability (Stilgoe et al., 2013; Kupper et al., 2015), which is implemented in the indicator system via indicator REF3. Critical reflection on values and motivations can also be seen as closely related to this (indicator REF4). Related RRI quality criteria include the need to explore underlying values in general (Wickson and Carew, 2014) as well as to recognize the shaping of decisions by personal values and scientific norms (Schuurbiers, 2011; Fraaije and Flipse, 2020). Finally, as part of the reflection process, it can also be seen as an aspect of RRI to become aware of existing uncertainties and limitations and to be able to understand the implications of them. In this context, Wickson and Carew (2014) speak of consciously identifying and considering both contextual and institutional limitations, which is subsequently captured via indicator REF5. Finally, the sixth indicator covers the degree of reflection with regard to the stakeholders to be included as well as the affected actors and individuals. Kupper et al. (2015) describe the implementation of an actor analysis as criteria of good RRI practice. Such an analysis aims at “identifying on whom the practice might have an impact or who might have an interest in, and might have relevant expertise for, the practice—and identifying how these actors relate to each other” (Klaassen et al., 2020, p. 228). The indicators and their descriptors are presented in Table 3 below. The descriptors used for the six indicators build on the work of Wickson and Carew (2014).

**Table 3:** Rubrics for reflection.

	**Low**	**Moderate**	**High**	**Very high**
**REF1** Critical reflection of problem definition	No procedure or declared interest in conducting reflective practice.	Informal, one-off or *ad hoc* process to examine the problem definition.	Occasional use of structured process for reflecting on problem definition.	Structured, purposeful periodic analytical review of problem definition.
**REF2** Critical reflection of assumptions, choices, and actions	No procedure or declared interest in conducting reflective practice.	Informal, one-off or *ad hoc* process to examine assumptions, choices and actions	Occasional use of structured process for reflecting assumptions, choices and actions	Structured, purposeful periodic analytical review of assumptions, choices, and actions.
**REF3** Critical reflection of responsibilities	No procedure or declared interest in conducting reflective practice.	Informal, one-off or *ad hoc* process to examine assumptions, choices and responsibilities.	Occasional use of structured process for reflecting responsibilities.	Structured, purposeful periodic analytical review of underlying responsibilities.
**REF4** Critical reflection of values and motivations	No procedure or declared interest in conducting reflective practice.	Informal, one-off or *ad hoc* process to examine underlying values and motivations among involved actors.	Occasional use of structured process for reflecting on underlying values and motivations among involved actors.	Structured, purposeful periodic analytical review of the underlying values and motivations among involved actors.
**REF5** Critical reflection of uncertainties and limitations	No procedure or declared interest in conducting reflective practice.	Informal, one-off or *ad hoc* process to examine uncertainties and limitations.	Occasional use of structured process for reflecting on uncertainties and limitations.	Structured, purposeful periodic analytical review of uncertainties and limitations.
**REF6** Analysis of relevant actors	No procedure or declared interest in analyzing potentially expertise-owning, affected and interested actors.	Informal, one-off or *ad hoc* process for the analysis of potentially expertise-owning, affected and interested actors.	There are consistent ideas about how to identify/analyze potentially expertise-owning, affected and interested actors and their relationship to each other.	Clear methodological approach for the analysis of potentially expertise-owning, affected and interested actors and their relationship to each other.

#### 3.3.3. Inclusion

Inclusion and deliberation are often considered synonymous to inclusive RTI discussion and negotiation processes that increasingly involve “new” voices in the governance and design of RTI (e.g., experts from the field, citizens, consumers, etc.) instead of top-down policies. A “broad, collective deliberation through processes of dialogue, engagement, and debate, inviting and listening to wider perspectives from publics and diverse stakeholders” (Owen et al., 2012, p. 755) can be seen as an essential goal of inclusion. The involvement of different actors should take place as early as possible, ideally already during the literature review phase (Pellé, 2016; Lubberink and Blok, 2017) and refer the formulation of the goals to be achieved with RTI. Inclusion and deliberation are particularly important among the RRI dimensions.

**Figure 3:**
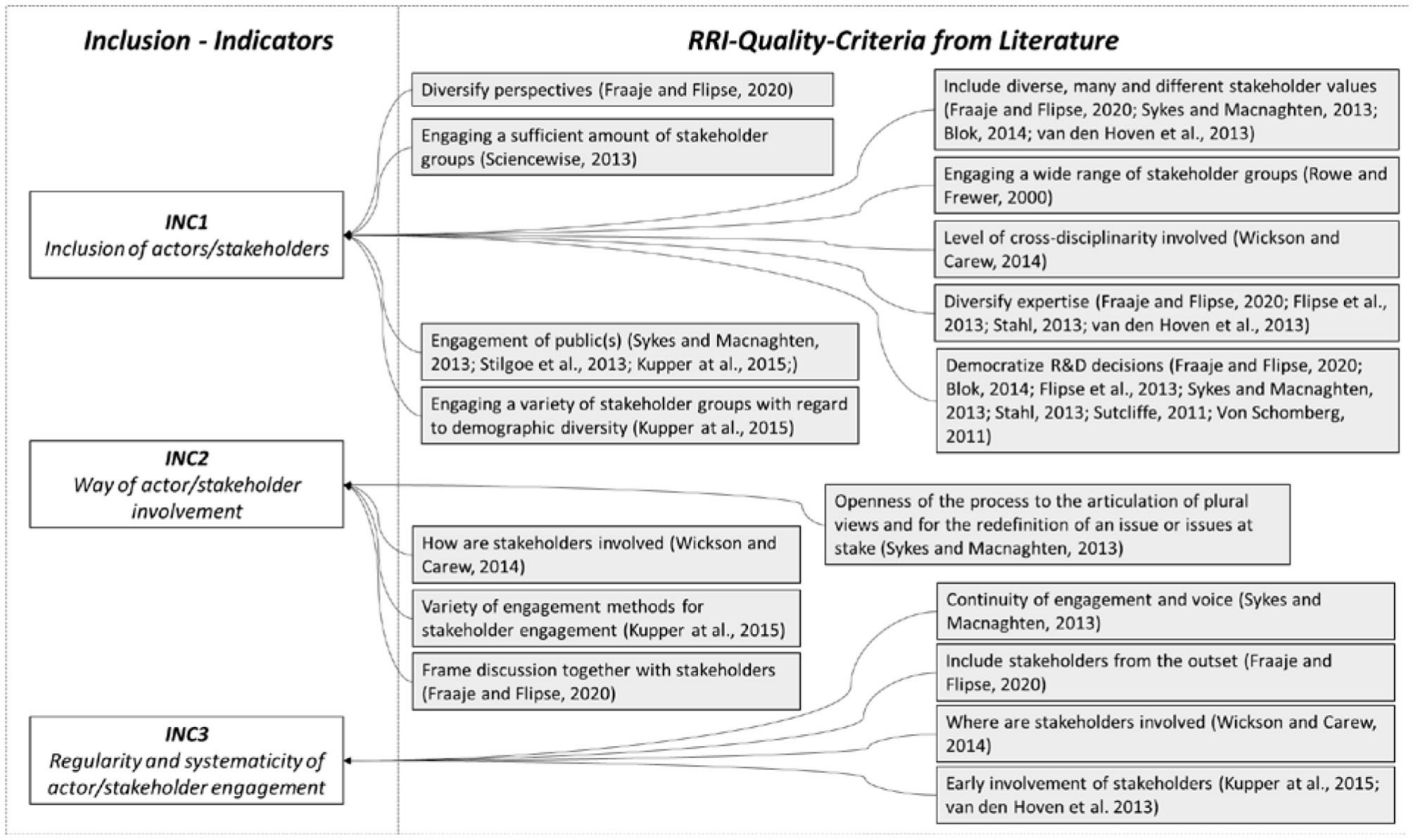
Quality criteria and indicators for inclusion.

The quality criteria identified in the literature with reference to inclusion and deliberation cover three central thematic fields and are shown in Figure 3. We hereby follow (Wickson and Carew, 2014, p. 263) who differentiate between (1) the “Level of cross-disciplinarity involved,” (2) “Where stakeholders are involved,” and (3) “How stakeholders are involved.” In more detail, indicator INC1 addresses the problem if the “relevant” stakeholders are involved in sufficient diversity and numbers. The high relevance of this indicator is also evident from the fact that related aspects are taken up very frequently in the literature (see Figure 3). In their review, Fraaije and Flipse (2020) refer to various publications that emphasize the need to include diverse stakeholder values (Sykes and Macnaghten, 2013; Van den Hoven et al., 2013; Blok, 2014) and perspectives (Fraaije and Flipse, 2020) as well as diversified stakeholder expertise (Flipse et al., 2013; Stahl et al., 2013; Van den Hoven et al., 2013). Consideration should also be given here to the inclusion of different (specialist) disciplines (Wickson and Carew, 2014), a potentially necessary demographic diversity (Kupper et al., 2015) and, in particular, the inclusion of the public (Stilgoe et al., 2013; Sykes and Macnaghten, 2013; Kupper et al., 2015). Against this backdrop, the descriptors used for the three indicators mirror the extent the aforementioned aspects are considered. Indicator INC2 additionally asks how and to what extent the actors/stakeholders are ultimately involved. Existing quality indicators mention the use of appropriate methods for stakeholder involvement (Kupper et al., 2015) as well as an ultimately joint design of the discussion with the stakeholders (Fraaije and Flipse, 2020) as elements to be considered in this regard. In a similar vein, Sykes and Macnaghten (2013) describe the openness of the process to the articulation of different views as an essential criterion for a good research process. Based on this, the indicator descriptors are designed to evaluate whether there are systematic efforts and convincing methods provided for stakeholder engagement, allowing them to contribute and discuss their own input. Finally, indicator INC3 refers to the regularity and systematicity of actor/stakeholder engagement. In this regard, the relevance of involving stakeholders from the outset (Fraaije and Flipse, 2020) or at least as early as possible (Van den Hoven et al., 2013; Kupper et al., 2015) is emphasized. This is mainly due to the fact that with the earliest possible involvement, there are also better opportunities to adapt the research direction (Flipse et al., 2013). Additionally, it is also occasionally noted that not only early, but also regular and ongoing stakeholder involvement is important (Sykes and Macnaghten, 2013; Wickson and Carew, 2014), which is also actively promoted in the research process (Wickson and Carew, 2014). In this context, Sykes and Macnaghten (2013) also see an essential quality criterion in how continuously the structured engagements in the research process take place and to what extent a continuity of voice is made possible.

#### 3.3.4. Responsiveness

Responsiveness addresses the problem of making responsible decisions and thus being able to control, for example to influence the innovation process (Owen et al., 2013; Lubberink and Blok, 2017; Fraaije and Flipse, 2020). In other words, responsiveness involves adapting to 'new' knowledge, e.g., about intended or unintended technological impacts while acknowledging the inadequacy of knowledge and control (Collingridge, 1980). This fourth dimension of RRI has close links to the previous three dimensions and describes the ability to respond to the outcomes from the activities of anticipation, inclusion, and reflexivity, and to adjust the direction of the RTI process accordingly.

**Figure 4:**
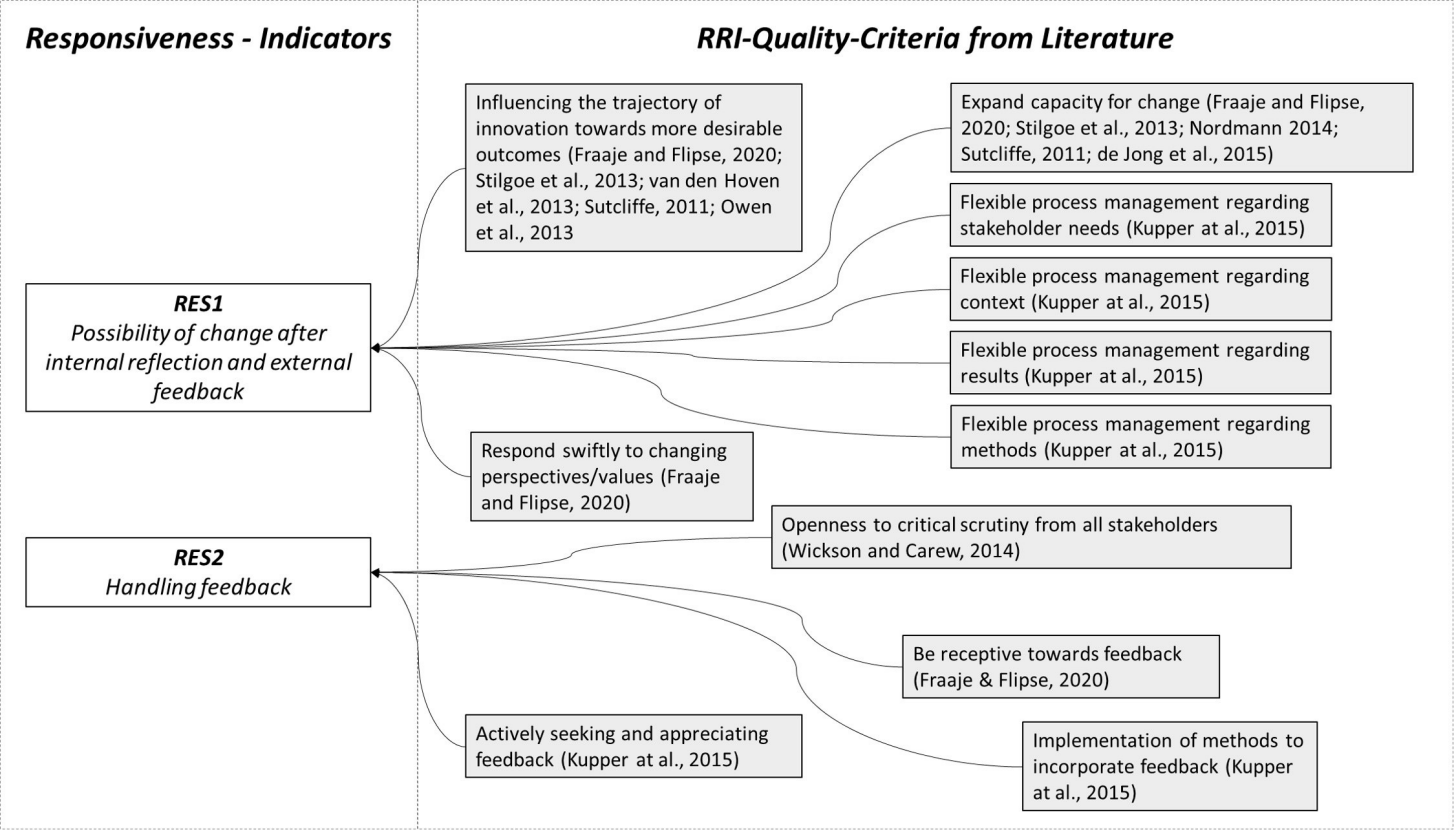
Quality criteria and indicators for responsiveness.

An essential aspect that can be found in various quality criteria in the literature is the ability or possibility of change after internal reflection and external feedback (Table 4). This is shown in Figure 4. Fraaije and Flipse (2020) for example cite the ability to react quickly to changing (societal) perspectives and values as a possible qualifier in this regard. Kupper et al. (2015) again mention the existence of flexible project management as a quality criterion to be considered in relation to responsiveness, which can ultimately also be seen as a way of expanding the 'capacity for change' increasingly cited in the literature (Owen et al., 2012; Flipse et al., 2013; Van den Hoven et al., 2013; Blok, 2014; Nordmann, 2014; de Jong et al., 2015). In principle, organizational and individual readiness to revise views and attitudes (Kupper et al., 2015) and to change role responsibilities (Stilgoe et al., 2013; Kupper et al., 2015) also fall into this space, although individual readiness in particular is likely to be difficult to assess in the context of an indicator evaluation. The descriptors for indicator RES1 are taken from Wickson and Carew (2014). The second indicator, RES2, complements the first indicator by examining how incoming feedback is handled and whether methods for taking it into account are implemented in the research process. In the RRI context, quality criteria for this include the general receptiveness to feedback (Fraaije and Flipse, 2020) and the existence of structures for obtaining and incorporating feedback (Kupper et al., 2015). In a sense, this reflects Wickson and Carew's (2014) question of openness to critical questioning and whether “the value of organized and disorganized skepticism [is] acknowledged” and conditions are “created to put it into practice” (Kupper et al., 2015, p. 28). Table 5 below shows the resulting rubrics for the dimension of responsiveness.

**Table 4:** Rubrics for inclusion.

	**Low**	**Moderate**	**High**	**Very high**
**INC1** Inclusion of actors/stakeholders	The involvement of stakeholders plays no or only a very minor role and is not further specified in terms of strategy and methodology.	Declared intention to involve various stakeholders on the basis of rather arbitrary or unspecified selection criteria.	Inclusion of the most relevant stakeholder groups based on specified selection criteria/methods and under consideration of the anchor points is foreseen.	Equal involvement of stakeholders in sufficient numbers and diversity, identified as relevant through specified selection criteria/methods and under consideration of the anchor points.
**INC2** Way of actor/stakeholder involvement	Purely informative stakeholder engagement without the possibility to discuss and give own input.	The methods used for stakeholder engagement allow stakeholder input without further discussion.	The methods used for stakeholder engagement allow for stakeholder input and limited discussion with/or between stakeholders.	The methods used for stakeholder engagement allow stakeholder input and a discussion with and between stakeholders.
**INC3** Regularity and systematicity of actor/stakeholder engagement	Communication with stakeholders takes only place toward the end of the research and innovation process.	Limited stages of the research and innovation process open for stakeholder engagement.	Inviting, incorporating, and integrating stakeholder views at various points along the research and innovation process.	Openly and actively seeking ongoing critical input, feedback and feed-forward from a range of stakeholders.

**Table 5:** Rubrics for responsiveness.

	**Low**	**Moderate**	**High**	**Very high**
**RES1** Possibility of change after internal reflection and external feedback	No evidence of potential for change in response to criticism/unsolicited feedback.	Stated willingness to accept change in response to internal reflective practice or external review and critique.	Clear indications of a capacity to adapt in response to reflective practice and external feedback.	Evidence of potential to adapt at a range of points in response to in-train reflective practice and external review/input/feedback.
**RES2** Handling feedback	Incoming feedback is not planned to be incorporated into the R&I process.	Incoming feedback is stated to be incorporated without clear methods or procedures.	There are consistent ideas about how to incorporate feedback.	Methods for incorporating feedback have been explored and implemented into the R&I process.

#### 3.3.5. Transparency

The dimension of transparency refers primarily to the role of the actors in the RTI advisory as well as decision-making process and the transparency that is created about this internally and externally, e.g., with regard to the question of the extent to which the actors will be able to influence decisions, or the question of how the contribution of the various actors is used and what effects this has in practice. Fraaije and Flipse (2020) point out a particularity with regard to the dimension of transparency compared to the four original dimensions formulated by Stilgoe et al. (2013). While anticipation, reflection, inclusion and responsiveness focus more on a “forward-looking” responsibility, transparency can be seen more as a “backward-looking” responsibility by “providing justification and clarity on decisions that were already taken” (Fraaije and Flipse, 2020, p. 120).

**Figure 5:**
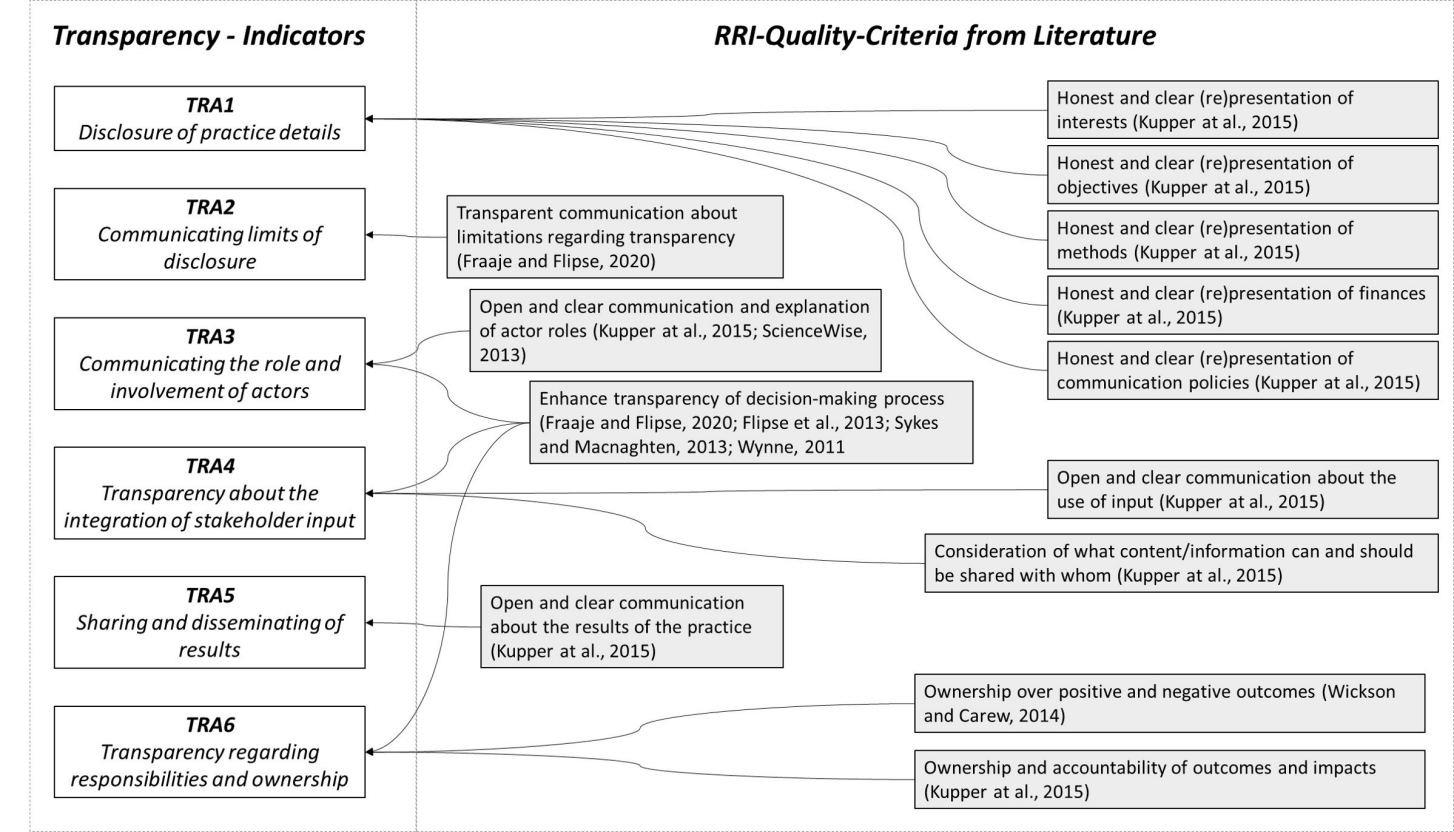
Quality criteria and indicators for transparency.

Although the dimension of transparency initially appeared “to be underdeveloped in RRI” (Forsberg et al., 2015), the literature provides various quality criteria, as shown in Figure 5. The criteria can be broken down into six groups, which are represented by the indicators shown in Table 6. Kupper et al. (2015) name as one of the essential initial steps in the area of transparency that practice details should be presented clearly and honestly. Accordingly, practice details include finances and methods as well as goals and interests. In particular, the literature increasingly refers to a transparent presentation of interests and backgrounds by asking about the underlying values (Sykes and Macnaghten, 2013) and assessment criteria (Fraaije and Flipse, 2020) as well as the ethical-social basis for decision-making (Flipse et al., 2013). Based on this, indicator TRA1 checks to what extent relevant information is published or to what extent such publication is envisaged in the course of the research process. Of course, it may well be that certain practice information cannot be made openly available, whether for legal or confidentiality reasons. Especially in such a case, it is then all the more important to transparently name and communicate these aspects and the reasons for confidentiality (Fraaije and Flipse, 2020). Indicator TRA3 focuses on the question of the extent to which transparent communication of the role and involvement of the actors involved in the research process is planned which, according to Sciencewise (2013), covers an essential aspect for the disclosure of deliberation and decision-making. In terms of transparency, however, it is not only important to make clear how actors are involved in the research processes, but ultimately also how the input of the stakeholders involved is dealt with and to what extent it is considered (Kupper et al., 2015). Indicator TRA4 therefore looks at the extent to which there are corresponding concepts or at least a stated willingness to take account of input received. Kupper et al. (2015) point out that sharing and publishing results is another quality criterion for transparency and emphasize that, in addition to the final results, the willingness to share preliminary and intermediate results is also of great value. Indicator TRA5 examines a corresponding commitment and the existence of a publication plan. Finally, indicator TRA6 looks at the extent to which there is clear and open communication with regard to delegation and ownership and thus refers to a quality aspect formulated by Wickson and Carew (2014) and also taken up by Kupper et al. (2015). The indicator descriptors are based on the formulations of Wickson and Carew (2014).

**Table 6:** Rubrics for transparency.

	**Low**	**Moderate**	**High**	**Very high**
**TRA1** Disclosure of practice details	There is no transparency of practice details. Neither with regard to objectives and methods nor with regard to finances and interests.	Some statement indicating the willingness to share information on objectives, methods, finances and interests is provided.	On most relevant aspects comprehensive information is provided.	Honest and clear representation of objectives, methods, financial means/expenditures as well as interests and affiliations of all actors.
**TRA2** Communicating limits of disclosure	No information about transparency concerning limitations and uncertainties.	Some statement indicating the willingness to share information on uncertainties and limitations.	Communication of uncertainties and limitations that are considered essential by the involved actors is envisaged.	A clear concept for communicating uncertainties and constraints that may be relevant to different stakeholders is presented.
**TRA3** Communicating the role and involvement of actors	No concept or statements on the role and involvement of relevant actors.	Stated willingness to inform about role and involvement of relevant actors without clear methods or procedures.	There are consistent ideas about how to inform about role and involvement of relevant actors.	A clear concept of how the role and involvement of relevant actors shall be communicated.
**TRA4** Transparency about the integration of stakeholder input	No concept or statements on how to disclose stakeholder input.	Stated willingness to disclose stakeholder input without clear methods or procedures.	There are consistent ideas about how to disclose stakeholder input.	A clear concept was developed on how to disclose stakeholder input.
**TRA5** Sharing and disseminating of results	No results are (planned to be) shared.	Results are (planned to be) shared only at one stage (preliminary, intermediate or final results).	A sharing of selected results with some involved and/or affected stakeholders is planned for different phases (preliminary, intermediate and final results).	A concept is in place that provides for an open provision of preliminary, intermediate and final results with the stakeholders involved and/or affected.
**TRA6** Transparency regarding responsibilities and ownership	Ownership of components and responsibilities are untraceable.	Indications of potential lines of delegation and ownership.	Established lines of delegation and ownership.	Openly communicated lines of delegation and ownership able to respond to process dynamics and contextual change.

## 4. Closing remarks and outlook

In this paper, we address the issue of an operationalization of the RRI concept by introducing an indicator system designed for supporting renewable energy innovation. The indicator system aims to help both the R&D funding organizations (government research funding and funding from private foundations) and R&D performing organizations (public and private) to apply the RRI concept. The indicator system presented here was developed as a toolkit to guide better design of innovations in the field of renewable energy and energy transition. Arguably, the indicator system can also be applied to other technological fields. It therefore represents a basis for further exploration and specification which can be realized, for example, by further adapting the indicator characteristics.

We started out by arguing that an operationalization of the RRI concept by means of an indicator system can contribute to better design research and innovation processes resulting in innovations that have a higher likelihood for being socially desirable and acceptable. This is particularly relevant for the case of Grand Societal Challenges, such as the energy transition process, for which technology plays a key role for creating a CO_2_-neutral society. In a first step, we collected RRI quality criteria based on an extensive review of the existing RRI literature. In a second step, the list of criteria was reviewed, grouped and bundled based on the five process dimensions of RRI (inclusion, reflexivity, anticipation, responsiveness, and transparency) in order to reduce the multitude of quality criteria to a manageable number. We finally developed a set of 20 basic indicators based on the emerging groups of quality criteria. For the indicators, a rubric system was chosen which mitigates the problems of subjectivity, measurability and specificity when applying RRI as a toolkit. In a next step, we will set up a guideline as well as an online tool for the application of the indicator system. We expect that the diffusion of the RRI concept can be fostered when it is linked to indicators and can be applied with a tool due to the inner functioning of bureaucratic institutions.

Several limitations of our approach to operationalize RRI in the context of supporting renewable energy innovation apply. First and foremost, it remains unclear whether the incentives to apply the indicator system are strong enough to prevent users from doing box-ticking. Consequently, we think that a clear communication of the advantages of the application is crucial. On the other hand, an indicator system can prevent funding organization from being satisfied with some sketchy sentences when they ask R&D projects to include RRI. Second, the degree of abstraction, and thus measurement of the indicators still represents a major challenge as also workshops with academic experts as well as practice experts conducted on the indicator systems have shown. The challenge of measurement is also linked to the fact that that there is no generally accepted definition of RRI and as it is a deeply normative concept (van de Poel, 2020). In a similar vein, one could argue that the way we, but also the users of the indicator system, understand RRI, and thus apply it through the indicator system is heavily influenced by our individual academic traditions, policy fields and political and cultural contexts (see also Doezema et al., 2019; Wittrock et al., 2021; Völker et al., 2023) which may represent a challenge when applying it across national borders or across academic fields.

## Data availability statement

The original contributions presented in the study are included in the article/supplementary material, further inquiries can be directed to the corresponding author.

## Author contributions

All authors listed have made a substantial, direct, and intellectual contribution to the work and approved it for publication.

## Footnotes

^1^This study is an outcome of the research project V4InnovatE. The project was funded by the German Federal Ministry for Economic Affairs and Climate Action (BMWK) under the funding scheme “Energy Transition and Society” within the 7th Energy Research Programme. V4InnovatE is the project acronym and stands for “Responsible innovation for the energy transition—development and implementation of an indicator system to increase the social acceptance of new technologies.” The joint project runs from October 2020 to September 2023.
